# Supplementary material for: Are Faba Bean and Pea Proteins Potential Whey Protein Substitutes in Infant Formulas? An In Vitro Dynamic Digestion Approach
Source: Foods. 2020 Mar 20;9(3):362. doi: 10.3390/foods9030362 (PMC7142966; doi:10.3390/foods9030362)
Supplement: Supplementary file 1 [file foods-09-00362-s001.pdf]

Table 1. Amino acid composition in the three protein ingredients: WPC (whey protein concentrate), PPC (pea protein concentrate) and FPC (faba bean protein concentrate). AA composition is expressed in mg amino acid / g protein. Essential amino acids (EAA) and non-EAA are expressed in % compared to the total AA. Data are means ± SD (n=2).

AA (mg/g protein)	WPC	PPC	FPC
Essential AA (EAA)			
Lys	115.9 ± 4.4	76.1 ± 2.1	60.3 ± 1.0
Tyr	41.9 ± 5.3	39.7 ± 3.6	30.3 ± 1.0
Phe	43.1 ± 3.6	55.6 ± 2.4	40.1 ± 1.8
Leu	141.0 ± 3.8	84.6 ± 2.5	69.5 ± 1.8
Ile	66.0 ± 1.7	48.4 ± 0.8	38.9 ± 0.7
Met	28.4 ± 4.5	14.1 ± 4.4	8.8 ± 2.5
Cys	31.5 ± 5.7	11.8 ± 5.6	12.6 ± 5.7
Val	61.2 ± 1.8	51.9 ± 1.6	41.5 ± 0.5
His	22.9 ± 2.9	24.6 ± 1.3	22.4 ± 0.8
Thr	59.5 ± 2.9	37.4 ± 3.5	31.7 ± 0.8
Non-EAA			
Arg	29.6 ± 3.0	88.2 ± 2.4	96.2 ± 1.2
Asx[1]	131.3 ± 8.7	120.4 ± 1.5	101.8 ± 1.0
Glx[2]	207.6 ± 8.2	182.5 ± 10.0	157.6 ± 8.6
Ser	52.7 ± 3.3	51.9 ± 3.1	45.1 ± 0.9
Pro	55.8 ± 5.0	43.2 ± 8.6	41.5 ± 3.2
Gly	20.3 ± 1.6	41.7 ± 2.0	37.8 ± 1.5
Ala	56.9 ± 2.1	43.9 ± 1.5	37.9 ± 1.2
Amino nitrogen	17.9 ± 1.0	17.9 ± 0.5	18.4 ± 0.4
EAA/ Total AA (%)	51.8 ± 0.4	43.0 ± 0.1	39.9 ± 0.8
Non-EAA/ Total AA (%)	48.2 ± 0.2	57.0 ± 0.3	60.1 ± 0.4

[1] Asx: aspartic acid + asparagine; [2] Glx: glutamic acid + glutamine
